# Why Work Overtime? A Systematic Review on the Evolutionary Trend and Influencing Factors of Work Hours in China

**DOI:** 10.3389/fpubh.2019.00343

**Published:** 2019-11-15

**Authors:** Bei Liu, Hong Chen, Xingxing Yang, Congmei Hou

**Affiliations:** School of Management, China University of Mining and Technology, Xuzhou, China

**Keywords:** Chinese employees, work hours, evolutionary trend, influencing factors, cross-temporal meta-analysis

## Abstract

**Objectives:** Research that examined changes in the laws in respect to work hours was of great importance for understanding its current status and causes. However, most research on work hours in China is still conducted using self-reported questionnaires, which lack coherence and depth, and are inadequate for exploring the evolutionary trend of work hours and its mechanism.

**Methods:** This study examined the evolutionary trend of Chinese employees' work hours by employing a cross-temporal meta-analysis, and employed the entropy weight method to analyze each influencing factor. The China National Knowledge Infrastructure (CNKI), VIP information and WanFang database were searched for Chinese-language peer-reviewed literature, and Google Scholar and the Web of Science were searched for related literature in the English language.

**Results:** A total of 36 pieces of literature were identified as having satisfied the quality standards for screening. The results indicated that work hours in China show a significant fluctuating upward trend. Most of the recent studies on work hours in China were cross-provincial investigations, and the issue of overtime among migrant workers has become the key focus of current research. Most studies on the work hours of Chinese employees were conducted in a manner whereby scholars played a leading role while the government assisted. Thus, government-led, intensive and nationwide research needs to be launched.

**Conclusions:** The issue of work hours in China should be taken seriously. The main influencing factors included survival indicators, such as labor market conditions and levels of medical security levels, followed by power-assisted indicators, such as personal income and distribution, while labor protection had a minimal impact. This study will contribute to a better understanding of the essence of work hours among Chinese employees, and will also help to provide a theoretical basis for further intervention study related to overtime work.

## Introduction

Work hours have always been the core of people's social life, and a certain amount of work hours was not only a necessary prerequisite for ensuring economic growth (1), but also an important variable that described current social institutional changes and productivity development (2). Focusing on system optimization and smart production, the Industrial 4.0 era has changed the mode of production and individual's way of life (3, 4), and the traditional manufacturing industry has begun to adopt the Internet of Things (which aims to connect humans, machines, and materials at any time or place) or big data analysis for intelligent transformation (5), which highlights the new characteristics for a changed reality. With high competition and uncertainty, this changing reality not only placed higher demands on management practice, but also underscored the urgency to elucidate the historical trend of work hours (6).

Therefore, research that examined changes in the laws in respect to work hours was of great importance for understanding its current status and causes. We may adopt the perspective of micro-macro to describe the current studies of work hours (7): The studies of work hours from a micro perspective mostly utilized cross-sectional data (8), and focused on the interaction between a specific variable and work hours during a specific period, such as exploring the relationship between overtime work, employee mental health (9), and organizational performance (10). The study of work hours from a macro perspective concentrated on the system of social structure, attempted to analyze the economic and social background of work hours, and constructed the position of work time in social development (11). Therefore, the study of work hours from a macro perspective was not limited to a specific individual or organizational variable, and its research perspective was extensive and comprehensive.

However, previous studies on work hours always focused on the micro level, and primarily concentrated on the number of work hours and the resulting variables. At the macro level, the evolution of work hours and the research on its influencing factors were relatively weak, which made it difficult to understand the research status and evolution of work hours, systematically and comprehensively. Moreover, most recent studies on work hours concentrated on developed countries (12), such as some European nations (13), and Japan (14). Studies have rarely been carried out on developing countries, and have failed to explore the influencing factors in developing countries. In fact, some progress has been made to limit work hours in several developed countries, such as the 405 Regulations issued by New York State (15) and the labor legislation reform in Finland (16). As for developing countries, obtaining authentic details about indigenous work hours and clarifying the impact mechanism were fundamental in order that they learned from the experience of developed countries, and organize their employees' work hours.

What's more, some studies have indicated that the work hours in developing countries were longer and that employees experienced more time-related stress (17), which would lead to more serious physical harm and psychological abnormalities (18).

While few studies have begun to explore the changes in work hours in developing countries, the results were not uniform. More researchers focus on India (19), Russia (20), and Latin America (21), while few studies focus on Chinese employees. As a typical large developing country, employees in China were suffering longer work hours and heavier workloads than those in Japan- the country which was once famous for long work hours (22), and the jargon, such as “karoshi (work to death)” also originated from Japan (23). Studies examined the relationship between work hours and occupational injuries and illnesses, and the results indicated that long work hours were a significant contributor to more serious occupational injuries (24). According to the resource loss theory and the resource finiteness theory, an individual's self-regulating resources are limited, and the continuous consumption process gradually leads to a decrease (25). Long work hours disturbed the rhythm of people's lives, which was harmful to individual development, and was detrimental to the long-term interests of the organization. Thus, it was important to pay close attention to Chinese employees' work hours. On the one hand, while considering both globalization and the development of information technology, the Chinese economy has entered a new normal period, and has achieved rapid increase over the past decades (26), which was the so-called “Chinese miracle (the continuous high-speed growth in China)” (27). Describing the evolution trend of Chinese employees' work hours and exploring its mechanism of impact may provide a new framework for understanding current national economic development. In particular, the study of its influencing factors was more conducive to the rational allocation of individual work hours, improving the efficiency of the use of national labor input, and thus promoting the development of the social economy. On the other hand, work hours were not only an important form of individual employment behavior and work ethics, but also an effective reflection of people's lifestyles and life concepts (28). The Chinese government has proposed a series of important strategic initiatives to improve people's quality of life, such as “Healthy China” and “Happy China” (29), which has challenged the concept of “work first” (30). Therefore, it was important to investigate the change in work hours and its mechanism of formation in order to balance the relationship between work and life, thereby promoting healthy and happy lives among people. Studying this issue also provided an important opportunity to analyze working behavior and the leisure life of Chinese employees.

In this study, the evolutionary trend were used to indicate the changing history and the future development trend of work hours of Chinese employees. However, current research on work hours in China is still at a preliminary stage because most studies continue to focus on investigating or comparing the specific number of work hours of different groups. Studies on its evolution trend were rare, and researches on its influencing factors were even more scarce, which contributes to the lack of coherence and depth in existing research on work hours in China. Therefore, our study aims to describe the evolution and trends in Chinese employees' work hours, and classify the influencing factors to calculate their weights in order to identify their impact on employees' work hours.

## Research Design and Method

### Cross-Temporal Meta-Analysis

The classical research paradigm employed the “time log method,” such that subjects recorded their activities over the course of a day or a fixed period of time (31). Researchers conducted a series of studies and analyses based on the reports of the subjects. However, most of the research objects were distributed in Western developed countries, and few research studies concentrated on the change in work hours in China, which means that previous research failed to provide a comprehensive and detailed analysis of current work hours in Chinese society. Twenge believed that the empirical researches on a topic in a certain period of time could be regarded as continuous and uninterrupted sampling, and she proposed a method of cross-sectional historical research based on the idea of longitudinal research designs by establishing a research sampling database that was established to describe the changing history of the topic over that period of time (32). Thus, this study conducted a unified quantitative analysis of the survey literature on work hours carried out by various Chinese scholars, in order to determine the changing trend in work hours among Chinese employees.

Referring to the principles of literature screening provided in Twenge's research, and the current research situation of work hours in China (33), the screening criteria for this study stipulated the following: (1) The research sample must represent a general Chinese employee group, and must include an employee group working in China; (2) The sample size, mean, standard deviation and other statistical results related to the measurement of work hours must be reported clearly in the study, and the sample size should be determined (although the data which were obtained from the China Population & Employment Statistics Yearbook did not provide the details of samples, and only included a type of official data released by the Chinese government, so such data were ultimately retained without including them in the sample size). No extremes were used in this study (by adopting the method of quartile; the screening results of the extremes of this study are shown in Supplementary Figure 1); (3) The research literature must clearly report the measurement criteria for employees' work hours. This study referred to the definition of work hours as defined by the International Labor Organization, i.e., work hours were directly or indirectly related to work; (4) As the purpose of this study was to describe the changing trend in work hours in China in a broader sense, this study does not set specific time ranges; (5) Special treatment should be given to some special situations, such as a certain articles containing survey data related to different groups, different years or different groups in the same year. Groups should then be divided into different groups and recorded by year. If the same article contains continuous tracking data of a certain group, the data related to the middle year should be selected and input. For data that tracks a group of employees over different years, the data were divided into years (i.e., a total of nine disassembled documents).

**Figure 1:**
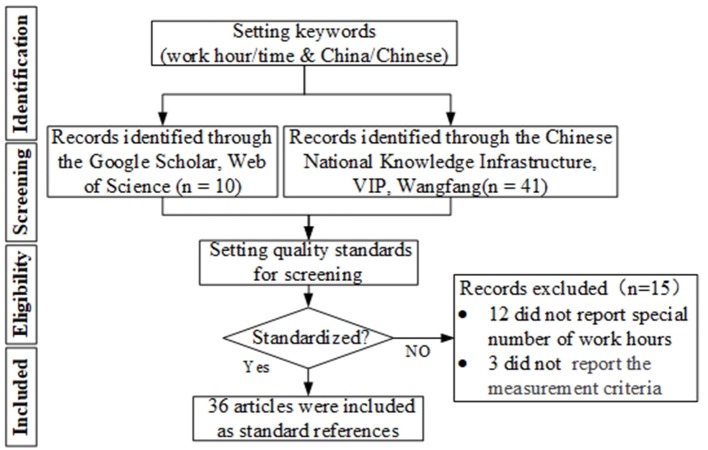
PRISMA diagram.

Our study followed the guidance outlined in Preferred Reporting Items for Systematic Reviews and Meta Analyses (PRISMA) (34), and a diagram of the search and selection process carried out in this study is shown in Figure 1 below.

As shown in Figure 1, this study collected data from Chinese journals, excellent masters, and doctoral dissertations (the literatures with high normalization, authority, and rigor) which were included in the China National Knowledge Infrastructure (CNKI), VIP information and WanFang database, as well as papers from Google Scholar and the Web of Science databases. In the process of retrieving data, this study did not specify specific research fields or a specific retrieval time for the literature search, so as to maximize the establishment of a research information database of work hours in China. Furthermore, we used “work hours” as search terms in Chinese journals, and used “China work hour,” “China work time,” “Chinese work hour,” and “Chinese work time” as search terms in foreign journals. In order to optimize the use of the information in each document, this study inputted information including industry distribution, regional distribution, average number, standard deviation, sample size, and data acquisition method into the database.

It should be noted that in some studies that did not report data related to the specific time period of the survey, we used the number of publication years “minus 2” as the research year (27). Some studies that did not provide standard deviation information were weighted according to the following formulas:

$$\begin{array}{l} \bar{x}=\Sigma x_{i}n_{i}/\Sigma n_{i}\\ S_{T}=\sqrt{\left[\Sigma {\text{n}}_{\text{i}}{s_{i}}^{2}+\Sigma n_{i}{\left(x_{i}-{\bar{x}}_{i}\right)}^{2}\right]/\Sigma n_{i}} \end{array}$$

Note :$\bar{x}$, S_*T*_, *n*_*i*_, x_*i*_, S_*i*_ represents the combined average, standard deviation, sample size, average, standard deviation.

### Data Resource of Influence Factors

One purpose of this study was to weight different influencing factors of work hours, and it was important to identify various influencing factors to achieve this goal. We carried out an extensive search for relevant studies on work hours in international databases and Chinese databases, and carefully screened each influencing factor. Furthermore, we rechecked and restocked the influencing factors constantly by inviting five experts of the management, organizational behavior, and sociology to retrieve related studies in different databases once again to re-examine the results.

Existing research on factors affecting work hours can be classified into two categories: The first category mainly focuses on the effects of an individual's motivation, living conditions, and other psychological factors related to work hours. For example, existing studies showed that individuals work longer hours to increase their income and alleviate financial pressure (35). The second type of factors largely reflect the influence of macro-indicators, such as current social development and individuals living standards, on individual work hours. Schor pointed out that individual wages, pensions, social insurance, vacation allowances, and other factors will affect the work hours of individuals, and even some marginal benefits in the organization were also identified as important factors that affect an individual's willingness to work a certain number of hours (36). Becker highlighted the impact of changes in income, pay, and working-hour productivity on work hours, and believed that an increase in work hours will also lead to an increase in these factors, though an increase in working-hour productivity will result in a continuous decline in work hours (37). Prasch's research suggested that empirical data related to the US in the 1970s showed that the stagnation of the US economy and the decline in wage rates led to an increase in the number of people who chose to increase their work hours (38). In conclusion, previous studies have found that wage receipts, vacation allowances at the organizational level, productivity levels, economic and market conditions, and social insurance at the social level are the main factors that affect work hours.

Comparatively, the conclusions that resulted from the first type of psychological influencing factors were relatively clear, and a one-way causal effect was identified between the influencing factors and work hours. However, the causal conclusions regarding the influencing factors of the second type of social indicators and work hours were vague, as were conclusions regarding the correlation analysis between the corresponding social indicators and work hours. Thus, the results highlighted that the relationship is not purely causal in nature. Moreover, the majority of existing research exploring the impact of social indicators on work hours focused on the causal relationship between a specific indicator (e.g., economic development and wage rate) and work hours, and ignored the mechanism of other factors in the relationship between them, which made it difficult to uniformly measure the weight of each indicator. Therefore, this study focused on the impact of the second type of social indicators on employees' work hours.

### Entropy Weight Method

In this study, the entropy weight method was employed to evaluate the weights of the influencing factors of work hours. The entropy weight method was an objective and comprehensive weighting method to measure the weights of indicators, and it has been widely adopted in many research areas, such as engineering technology, social economy, and social science (39, 40). The weights were determined by measuring the amount of information transmitted to decision makers by the indicators (41). Generally speaking, the greater the impact utility of an index, the greater the weight in the comprehensive evaluation (42). Therefore, using the entropy weight method to evaluate the influencing factors of work hours would not only allow us to obtain the weight coefficients of each evaluation index, but also to compare the effects of various indicators. The specific steps are as follows:

(1) The data of the indices were standardized to eliminate the dimension difference. The specific methods were as follows: Positive index:
  $$\begin{array}{l} p_{ij}=\frac{X_{ij}-X_{i,\min}}{X_{i,\max}-X_{i,\min}} \end{array}$$
  Negative index:
  $$\begin{array}{l} p_{ij}=\frac{X_{\text{i},\max}-X_{ij}}{X_{i,\max}-X_{i,\min}} \end{array}$$
  Among them, *P*_*i*_ represented the standardization coefficient of index *i*, the value of index *X*_*ij*_ in year *j*, and *X*_*i, min*_ and *X*_*i, max*_ represented the minimum and maximum value of index *i* during the study period.
(2) In the specific data processing process, this study assumed that there were m samples of work hours collected in a total number of years (*m* = 17), and there were n factors that affected work hours, *X*_*i*_ (*i* = 1,…, *n*), respectively, so as to standardize the variables to calculate the information entropy of the impact indicators. The entropy *F*_*i*_ of the second index can be defined as:
  $$\begin{array}{l} F_{ij}=-k\overset{n}{\sum }H_{ij}\ln\;H_{ij}\\ H_{ij}=\frac{p_{ij}}{\underset{j}{\sum }p_{ij}} \end{array}$$

*P*_*ij*_ represented the standardization coefficient of index *i* in year *j*, and *K* = $\frac{1}{\ln\;m}$. After the index entropy value is determined, the entropy weight *W*_*i*_ of the first index is determined according to the following formula:

$$\begin{array}{l} W_{i}=\frac{1-F_{i}}{n-\overset{n}{\underset{i=1}{\sum }}F_{i}} \end{array}$$

## Results

### Descriptive Statistical Analysis

Based on the results of the literature search and the general steps involved in the cross-sectional historical research method, this study coded the literature with the results of the retrieval, and supplemented the missing values of the database with the help of formulas 1 and 2. Among these, 36 documents satisfied the criteria, i.e., 31 documents in Chinese and five in English. Of the 36 documents collected, 11 were divided into different data strips. Therefore, 51 datasets were obtained in this study, covering more than 7,800 participants. Table 1 provides a detailed description of the coding types of the collected samples.

**Table 1:** Selected publications for review.

**N**	**Author**	**Year of survey**	**Title**	**Group**	**Sample size**	**Region**	**Types of survey**	**Data type**
1	Shen et al. (43)	2009	An empirircal study on the influencing factors of faculty working time	Faculty	4,200	M	Self-conducted	*C*
2	Tian (44)	2009	The effect of health status and health shocks on working hours	Worker aged above 45	633	M	Self-conducted	C
3	Cheng et al. (45)	2009	Working hours, leisure time and urban employment of farmer migrant workers: Emprise analysis on 1446 survey samples in Shanghai.	Migrant worker	1,446	E	Self-conducted	C
4	Yang et al. (46)	2011	The impact of migrant workers' income and working hours on life satisfaction	Migrant worker	310	N	Self-conducted	C
5	Liu (47)	2013	A study of university teachers' working time	Teacher	358	M	Self-conducted	C
6	Jin et al. (48)	2014	Conflict of work hours and work family: A study based on sex difference	General staff	2,030	M	Self-conducted	G
7	Zhang (49)	2011	A study on the impact of employees' working hours on job satisfaction, organizational commitment and turnover tendency.	General staff	883	M	Self-conducted	C
8a	Wu (50)	2015	Impact of hours worked on occupational well-being-An empirical analysis based on three typical occupations	Industrial worker	234	M	Self-conducted	G
8b		2015		Migrant worker	687			
8c		2015		Civil service	358			
9	Wang and Meng (51)	2012	Distribution of the working hours among primary care staffs	Primary care staffs	863	M	Self-conducted	G
10	Qin et al. (52)	2017	Research on teaching time structures of the compulsory education teachers-Based on the data of 20 cities/counties from 10 provinces in China	Teacher	2,018	M	Self-conducted	C
11	Tong (53)	2014	A study on the working time of junior middle school teachers and its influencing factors -An analysis based on China's Educational Follow-up Survey (CEPS)	Teacher	1,136	M	Project-conducted	C
12a	Zhu and Jiang (54)	2009	Research on the work-time effect of social insurance-Evidence from CHNS Data	General staff	2,190	M	Project-conducted	G
12b		2011			2,235	M		
13	Guo and Zhang (55)	2007	Intergenerational differences in working time of migrant workers: Changes in labor supply behavior of migrant workers in the process of urbanization	Migrant worker	6,551	M	Project-conducted	G
14	Zhai (56)	2016	Research on the impact of teaching time of teachers in rural areas on their work enthusiasm -Based on survey data from teachers in D county of J province	Teacher	251	M	Project-conducted	D
15a	Qiu (57)	2007	The adjustment of minimum wage effects on employment and working time-based on DID model	Migrant worker	2,020	M	Project-conducted	D
15b		2008		Migrant worker	2,899	M		
16	Gao (58)	2007	Research on the relationship between demographic factors and working time allocation model of managers	Manager	294	N	Self-conducted	D
17	Qi et al. (59)	2010	Is there a second shift phenomenon for women-An empirical study based on women's education, profession, and income characteristics.	General staff	4,582	M	Self-conducted	*C*
18	Spector et al. (60)	2004	A cross-national comparative study of work-family stressors, working hours and well-being: China and Latin America versus the Anglo World	Manager	768	HMT	Self-conducted	C
19a	Peng (61)	2005	Employment and working hour effects of minimum wage increase: Evidence from China	General staff	1,622	M	Project-conducted	G
19b		2006			1,731			
20	Lu (62)	2005	Working hours and personal preference among Taiwanese employees	General staff	1,122	HMT	Project-conducted	C
21	Yamashita et al. (63)	2010	Are East Asians happy to work more or less? Associations between working hours, relative income and happiness in China, Japan, South Korea and Taiwan	General staff	3866	M	Project-conducted	C
22a	Dong and An (64)	2008	Gender patterns and value of unpaid care work: findings from China's first large-scale Time Use Survey	General staff	18,215	M	Project-conducted	C
22b		2003			18,927			
23	NBSC (65)	2002	China Population & Employment Statistics Yearbook	General staff	No report	M	National-conducted	R&Y
24	NBSC (66)	2003	China Population & Employment Statistics Yearbook	General staff	No report	M	National-conducted	R&Y
25	NBSC (67)	2004	China Population & Employment Statistics Yearbook	General staff	No report	M	National-conducted	R&Y
26	NBSC (68)	2005	China Population & Employment Statistics Yearbook	General staff	No report	M	National-conducted	R&Y
27	NBSC (69)	2006	China Population & Employment Statistics Yearbook	General staff	No report	M	National-conducted	R&Y
28	NBSC (70)	2007	China Population & Employment Statistics Yearbook	General staff	No report	M	National-conducted	R&Y
29	NBSC (71)	2008	China Population & Employment Statistics Yearbook	General staff	No report	M	National-conducted	R&Y
30	NBSC (72)	2009	China Population & Employment Statistics Yearbook	General staff	No report	M	National-conducted	R&Y
31	NBSC (73)	2010	China Population & Employment Statistics Yearbook	General staff	No report	M	National-conducted	R&Y
32	NBSC (74)	2011	China Population & Employment Statistics Yearbook	General staff	No report	M	National-conducted	R&Y
33	NBSC (75)	2012	China Population & Employment Statistics Yearbook	General staff	No report	M	National-conducted	R&Y
34	NBSC (76)	2013	China Population & Employment Statistics Yearbook	General staff	No report	M	National-conducted	R&Y
35	NBSC (77)	2014	China Population & Employment Statistics Yearbook	General staff	No report	M	National-conducted	R&Y
36	NBSC (78)	2015	China Population & Employment Statistics Yearbook	General staff	No report	M	National-conducted	R&Y

E, Eastern China; N, Northeast China; HMT, Hong Kong, Macao and Taiwan; M, Two or more categories were included; C, Core (included SSCI/SCI/CSSCI).The articles that were peer-reviewed publications, and were indexed in the Social Sciences Citation Index or Sciences Citation Index or Chinese Social Sciences Citation Index; G, General. The articles that were not indexed in the Social Sciences Citation Index or Sciences Citation Index or Chinese Social Sciences Citation Index; D, Dissertations or collections; R&Y, Reports, yearbooks.

*“Self-conducted”: The research activities in such articles led by scholars*.

*“Project-conducted”: The research carried out in such articles commissioned by the government*.

*“National or international-conducted”: The research carried out in such articles led by the government*.

**Figure 2:**
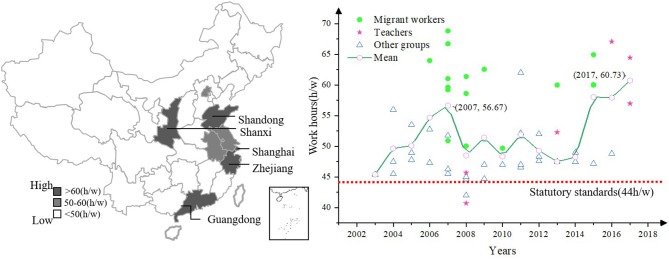
The chronological clues and geographical distribution of the work hours in China.

Similar as the Sciences Citation Index (SCI) and the Social Sciences Citation Index (SSCI), the Chinese Social Sciences Citation Index (CSSCI) was commonly used to retrieve academic literature related to the humanities and social sciences in China, and its representativeness and applicability have been widely recognized in China (79). Thus, this study referred to “core journals” to identify literature that was indexed in the SCI, SSCI, and CSSCI, and “general journals” to identify literature that was not included in the aforementioned indexes (80). As shown in Table 1, the preliminary coding results show that at present, in China, most of the literature on work hours was published in the “general” level, while related research studies published in the “core” level were limited. We classified the literature into three types to categorize the research on the basis of their funding (i.e., independently carried out, project-conducted, and national or international-conducted). The results show that most of the current research on the work hours of Chinese employees were scholar-led and government-assisted. Therefore, nationwide and intensive government-led research needs to be launched. In addition, most of the recent studies on work hours in China conducted cross-provincial investigations, especially in Guangdong, Zhejiang, where employees work longer hours. Figure 2 depicted the chronological clues and geographical distribution of work hours among various groups in China.

To our knowledge, our study contained the longest time-span of Chinese work hours of existing literatures. The study found that the work hours of Chinese employees showed a fluctuant upward trend, and the average weekly work hours of the sample group were 52 h/week (*SD* = 7.98), which was significantly higher than the legal benchmark of 44 h stipulated in the Labor Law of the People's Republic of China (2009). According to the attributes of the samples, the selected literature may classify into two types: One included “general staff groups” (the study did not report the particular occupations of the samples, which represented 69% of the total sample), and the other type included “special working groups” (the study reported the specific occupations, such as teachers, doctors, and managers, or social identity, i.e., migrant workers, of the samples, which represented 31% of the total sample). Specifically, the group of migrant workers referred to those who work in cities and hold rural household registrations (81), and their work hours were higher than those of any other groups.

### Homogeneity Test

During the preliminary description stage of the sample, the study found that the work hours of special working groups (e.g., migrant workers, teachers, etc.) in China were significantly higher than those of the general group. In this study, the work hours of general and special groups of employees were treated as opposite groups. A data homogeneity test was conducted to determine the selection of the fixed-effect model and random effect model for use in the meta-analysis. The results of the independent sample *t*-test showed that the variance of work hours variables between general and special groups was homogeneous (*F* = 13.718, *P* < 0.05). A significant difference was found between the two groups (*t* = 5.156, *P* < 0.05), and the variance was homogeneous.

### Analysis of the Time-Effect of Chinese Employees' Work Hours

After a significant correlation between the samples and years was obtained, the data processing method, which was used by previous researchers, was employed to fit the linear regression model by taking the number of work hours as the dependent variable, age as the independent variable and weighting the sample size (31). The results of the weighted regression analysis showed that the regression equation between years and work hours is *y* = 0.482*x* − 917 (0.482 is the non-standardized regression coefficient, *x* expresses age, −917 is the constant term, *y* represents the work hours of Chinese employees).

By substituting the years 2000 and 2020 into the regression equation, the average work hours of Chinese employees in 2000 and 2020 were *M*_2000_ (47) and *M*_2020_ (56.64), respectively. Moreover, the following measures were taken to avoid the emergence of ecological fallacies (82). On the one hand, when formulating a regression equation between sample variables and years, the sample size was weighted. On the other hand, in the process of calculating the change in the work hours of Chinese employees over the past 20 years, data, such as the sample's average score and standard deviation, were obtained from the scores of the variables reported in the weighted sample (see Table 2).

**Table 2:** The time-effect variation of work hours.

**Variable**	**Year**		**Variations**				
	**β**	***R*^*2*^**	***M*_*2000*_**	***M*_*2020*_**	***M*_*change*_**	***SD***	***d***
Average weekly work hours	0.482**	0.092	47	56.64	9.64	19.64	0.50

** *P < 0. 01; M_change_ = M_2020_−M_2000_, d = (M_2020_−M_2000_)/SD*.

**Table 4** provides a detailed illustration of the changes in the age utility of the work hours of Chinese employees. According to Cohen's distinction of the magnitude of effect (absolute value), an absolute value of effect <0.5 is a “small effect” whereas between 0.5 and 0.8 indicates a “medium effect,” and >0.8 suggests a “big effect” (83). According to this standard, during the past 20 years, the change in the work hours of Chinese employees reached a level that warrants attention.

### Construction of the Influencing Factors Model of Chinese Employees' Work Hours

To our knowledge, most previous studies concentrated on the consequences of work hours, and research on its influencing factors were relatively limited. This study summarized the influencing factors of work hours, and collated them in Table 3.

**Table 3:** Combing macro-impact indicators of work hours.

Personal Income and Domination	Income	Income is the tangible result of work hours, and it can promote the happiness of work hours (35).
		Wages can effectively regulate the relationship between unemployment and work hours (84).
	Domination	Whether it involves an acquisition-spending lifestyle or a high-consumption-intense-working lifestyle, consumption is closely related to work hours (85).
Economic Development and Motivation	Economic Development	Economic stagnation has led to more people choosing to work longer hours (38).
	Innovation ability	R&D personnel will work longer hours in order to improve the level of scientific and technological innovation (86).
		Innovation has broken the original job placement paradigm, resulting in unemployment or the re-employment of some employees (87).
		The rapid popularization of mechanized production replaces traditional handicraft production, making individual work hours more flexible (88).
Labor market	Supply-demand relationship	Competitive pressures, the threat of unemployment, and other factors will encourage individuals to work longer hours (89).
	Labor Disputes and Injuries	Physical injuries caused by occupational illnesses, such as pain, can lead to absenteeism, reduced work efficiency, and even withdrawal from the labor market, which can reduce the working time of individuals (90).
Medical and health security	Medical security	If individuals can enjoy medical and health benefits, they will reduce their labor input accordingly (91).
Social insurance	Insurance coverage	Workers who enjoy old-age insurance are more likely to work shorter hours (92).
		Wives whose spouses are uninsured are more likely to work full-time, because wives whose spouses are uninsured are more likely to request a job with health benefits and usually have to work full-time to obtain those benefits (91).
		The unemployment insurance program started in Canada in 1997 and has effectively reduced the work hours of some overtime workers (93).

Based on Table 3, this study developed a macro-influence factor model of work hours, which contained five types of factors that determine work hours, i.e., personal income and distribution, labor market conditions, economic development level and labor productivity, social insurance status, and medical insurance level. In order to quantify those factors, we selected different indicators to weight them. The model indicators are shown in Table 4.

**Table 4:** Index system of influencing factors of Chinese employees' work hours.

**Primary indices**	**Secondary indices**	**Unit**	**Primary indices**	**Secondary indices**	**Unit**
Personal Income and domination	Consumption level	*Yuan*	Medical and health security	Personal burden ratio of social health expenditure	%
	Per capita income ratio between urban and rural	%		Number of beds in health institutions	*Ten thousand*
	Urban household income	*Case*		Annual self paid health expense	*Yuan*
	Rural household income	*Case*			
Economic development and motivation	Per capita gross national product	*Yuan*	Labor market	Employment rate	%
	External technology dependence	%		Unemployment rate	%
	Full-time equivalent output R&D	*Items/Ten Thousands*		Employed population	*Ten thousand*
	Turnover of technology market	*Million*		Number of occupational diseases	*Case*
	Acceptance number of patent application for invention	*Piece*		Number of labor disputes	*Piece*
Social insurance	Unemployment insurance coverage				%
	Industrial injury insurance coverage				%
	Medical insurance coverage				%

According to the constructed evaluation index system, this study calculated the score of each index based on data obtained from the China Economic Yearbook, China Statistical Yearbook, and China Labor Statistics Yearbook, as well as from their websites, for the period 2000–2016. Based on the relevant research data, this study obtained data related to 51 work hours for Chinese employees, covering more than 7,800 participants. A total of 20 statistical datasets related to the evaluation indicators of influencing factors were obtained, and the original data matrix was established. Following standardization, the standardized results were substituted into the formula to calculate the information entropy *F*_*i*_ of each index. Finally, the entropy weight *W*_*i*_ of each index was obtained by substituting the *F*_*i*_ of each index into the below formula. According to the results of the correlation analysis, the weight and ranking of each evaluation index are shown in Table 5.

**Table 5:** Weight of influencing factors on work hours of Chinese employees.

**Primary indices**	**Secondary indices**	**Weight**	**Positive/negative**	**Sort**
Personal income and domination	Consumption level	0.062	*P*	2
	Per capita income ratio between urban and rural	0.032	*N*	8
	Urban household income	0.021	*P*	14
	Rural household income	0.019	*P*	16
Economic development and motivation	Per capita gross national product	0.023	*P*	13
	External technology dependence	0.025	*P*	11
	Full-time equivalent output R&D	0.012	*P*	19
	Turnover of technology market	0.016	*P*	17
	Acceptance number of patent application for invention	0.015	*P*	18
Labor market	Employment rate	0.024	*P*	12
	Unemployment rate	0.050	*P*	5
	Employed population	0.428	*P*	1
	Number of occupational diseases	0.033	*P*	7
Medical and health security	Personal burden ratio of social health expenditure	0.031	*P*	9
	Number of beds in health institutions	0.020	*P*	15
	Annual self paid health expense	0.051	*P*	4
	Personal burden ratio of social health expenditure	0.057	*P*	3
Social insurance	Unemployment insurance coverage	0.027	*P*	10
	Industrial injury insurance coverage	0.009	*P*	20
	Medical insurance coverage	0.045	*P*	6

First, from the point of view of the weight distribution of the impact indicators of the work hours of Chinese employees, the macro indicators had an important impact on the work hours of domestic employees, and included labor market conditions such as the employment/unemployment status of employees, labor disputes, and other related factors. Other impact indicators, such as the proportion of the financial burden arising from social healthcare expenditure, per capita health expenditure, the number of occupational illnesses, and other factors that reflected the level of individual medical security, also had a profound impact on work hours. Second, it reflected the effect of income, consumption, and other indicators of personal income and the distribution of work hours. At the same time, in the case of poor protection under labor rights in China, the weights of social insurance coverage, medical expenses, and other indicators obtained by the entropy weight method were lower, especially the lowest weight of industrial injury insurance coverage indicators.

## Discussion

### Insight Into the Evolution of Chinese Employees' Work Hours: Overtime Work and “Medium Effect”

The results of the analysis, which were obtained by using the cross-sectional historical research method, showed that the evolution trend of Chinese employees' work hours in recent years was not optimistic, and overtime work among employees is common. On the one hand, the current work hours of Chinese employees are fluctuating: In 2017, for example, the average work hours of the sample group were 60.73 h per week, which exceeded the Chinese Labor Law legal standard of work hours (i.e., 44 h per week) by 38%, which was even greater than the labor standards set by the International Labor Organization (ILO) which stipulate that work hours should not exceed 40 h per week. The prediction based on the historical research method of cross-cutting showed that the work hours of Chinese employees will continue to rise in 2020. On the other hand, according to Cohen's criteria for dividing the amount of effect (absolute value) (46), the variation in the work hours of Chinese employees has reached the level of “medium effect,” which should be taken seriously. The results furthermore indicated that the issue of overtime work among Chinese employees needs urgent attention.

Long work hours are one of the characteristics that are commonly observed in most countries that are experiencing a process of rapid economic development. At some stage, almost every country's work hours show a trend that shifts from rapid growth to a gradual decrease (94). In developed countries, in particular, such as the UK and the US, the trend from high to low is more obvious. During the First Industrial Revolution, a large number of employees worked overtime, and reducing the number of hours worked became one of the main goals of the workers' movement (95). After the Second Industrial Revolution, work hours began to decrease and fluctuate in developed countries (96). China's current work hours are still in the growth phase, and the peak has not yet been reached. Therefore, from the perspective of work hours, while China's economy has developed rapidly in recent years and its Gross National Product (GNP) has continued to grow, its level of development and people's quality of life still lag far behind those of developed countries, such as the UK and US.

### Geographical Distribution of Typical Research Objects of Work Hours in China: Migrant Workers in Southeast Coastal Areas

Overtime work is common in the eastern coastal areas of China, especially in Guangdong and Zhejiang. The southeastern coastal areas of China have a relatively high level of economic development (97). The Pearl River Delta and the economic belt of Jiangsu, Zhejiang, and Shanghai are the leading regions in China. In this study, the overtime work of employees from Guangdong and Zhejiang also reflects the positive correlation between the regional economic development level and individual work hours. In the high incidence areas where these employees work overtime, the problem of the Chinese migrant workers group is most prominent. Migrant workers refer to those who have a rural household registration status, but work in cities and towns (98).

Compared with urban employees, the work of migrant workers in China is highly mobile and very intense (99, 100). Workers lack legal protection and are marginalized, which is typical the work of “informal workers” (101). Studies have shown that the current multi-segmentation of China's labor market is the main reason for the unfair employment of migrant workers (102). Given this separation, rural laborers are not free to enter the urban labor market, and wage discrimination and employment discrimination are widespread. Therefore, most migrant workers can only engage in traditional industries that require more physical labor, such as the construction industry (103). In addition, the relevant system of safeguarding the labor rights and interests of migrant workers in China has not yet been perfected (104), which leads to exploitation by employers in respect to the work hours of migrant workers.

### Analysis of the Influencing Factors of Work Hours: Survival Needs and Social Needs

The weight analysis of the factors affecting the work hours of Chinese employees showed that labor market indicators, such as the employment rate and unemployment rate, were the main factors driving the growth of employees' work hours, followed by factors related to medical and health care, such as the individual medical expenditure and occupational illnesses. According to Maslow's hierarchy of needs theory, survival needs and security needs are at the lower level of an individual's demands, and can more effectively mobilize an individual's behavior (105). In this study, the labor market indicators, based on the employment rate, and medical security factors, based on individual health expenditures, represent survival indicators that maintain the basic survival and safety needs of individuals (106, 107).

On the one hand, the positive correlation between the employment rate, the unemployment rate, and work hours indicates that increasing the number of work hours is an important means by which individuals can avoid the pressure associated with unemployment, allowing them to ensure their survival. Studies have shown that work hours are an implicit way of regulating employment pressure. Employees reduce or increase their work hours in exchange for their initiative to exit or enter certain labor markets (108). On the other hand, the important impact utility of medical and health care factors, such as individual health expenditure, on individual work hours, which are used to guarantee the basic personal safety of individuals, also suggests that increasing the number of work hours is an effective way by which individuals can pay medical expenses and safeguard their health. Therefore, in general, the dominant position of these survival indicators indicates that the demand for long work hours by most Chinese people is still at a stage of rigid demand, such that employees seek to maintain their employment and ensure their physical safety.

From the above process, factors such as an individual's consumption expenditure can also lead to a significant increase in work hours. Compared with the survival indicators, such as labor market indicators and medical security, individual consumption and income indicators belong to a higher level of social indicators. In this type of hierarchy, the main reasons that an individual seeks to extend their work hours change from a desire to maintain their existing job to a desire to obtain a wage increase and protect their consumption needs. There is also a shift from physiological needs to the furtherance and enjoyment of social needs. Schor pointed out that the cause of an individual's excessive work was the “work-consumption circle.” She believed that with economic development, individual psychology, in terms of comparing one's consumption habits with those of others, becomes more prominent, and that wasteful lifestyles, which center on individual-consumption, gradually become more commonplace. This psychology urged individuals to increase their work hours for consumption purposes (109).

Indicators that reflect the level of national scientific and technological innovation, such as the number of patents and patent applications for inventions, have positive effects on work hours, after factors such as the labor market and level of personal income. Theoretically, technological progress, as well as the improvement in management brought about by technological progress, can enable organizations to produce the same amount of homogeneous products with fewer production factors than before (110, 111), thus promoting an improvement in organizational labor productivity and a reduction in the individual work hours of employees. However, the results of this study reflected the positive relationship between them. We believe that the continuous advancement of modern science and technology, especially information technology, has accelerated the operation of business, intensified time competition (112), further blurred the boundaries between the individual's work space and living space (113), and led to an indefinite extension in the number of employee work hours. Thus, work hours have not decreased, either overall or at an individual level.

Studies have shown that policy constraints in respect to government labor protection are an important means of reducing individual work hours (15, 16). The results of this study reflected the low impact of social insurance factors. We believe that there are two possible reasons for this finding: On the one hand, there is a lack of standardization in China's market relations, an urgent need to improve the government's protection system for labor rights and interests (114), and a failure by some organizations to comply with the corresponding work hours regulations, and such organizations continue to demand overtime among their employees (115). On the other hand, from the perspective of values, the Chinese cultural background is more inclined to collectivism (116), and the power distance between employees and leaders is relatively low (117), which leads Chinese employees to comply with the overtime demands of their employers, even in the absence of corresponding compensation. These factors mean that the relevant measures associated with labor protection rights cannot have a significant impact on employees' work hours. Therefore, when the entropy weight method is used to calculate the impact weights, the weight coefficient will be lower.

## Limitation

This research strived to be scientific and rigorous during the process of selecting literature and conducting the quantitative research. However, some limitations and biases should be considered when interpreting this study. There may be a language bias because only publications in Chinese or English were included in our database, and it is possible that some studies could not be assessed for their eligibility simply due to the constraints posed by the language of publication (not in English or Chinese). Although this study constructed a database of Chinese employees' work hours by referring to existing research with the largest time span, strictly speaking, this database was not very suitable for time series analysis due to its limited data and time span. Thus, the temporal relationships among variables need to be further confirmed. Last, individual behavior is influenced by many factors. Our influencing factors derive from existing research, which may address all of the factors that affect work hours, and the influencing factors of work hours need to be further explored.

## Conclusion

The changing trend in employees' work hours in China is on the rise, and overtime work among employees is widespread. The change in the trend over the past 20 years has reached the level of a “moderate effect,” which warrants attention. Overtime work is common among employees in eastern coastal areas such as Guangzhou and Zhejiang. In such areas, overtime work among migrant workers is the most prominent.

The main factors that prompt Chinese employees to work overtime include survival indicators, such as the labor market and medical security, followed by social indicators, such as individual income and distribution, while labor protection factors, such as social insurance, have the least impact on an individual's work hours.

### Policy Suggestions and Enlightenment

This study revealed the evolutionary trend of employees' work hours in China in recent years. The results showed that, at present, the number of employee work hours was generally higher than the statutory standard, and the phenomenon of overtime work among migrant workers, in particular, was the most serious issue. Statistical results, that are based on effectiveness, further highlighted the urgency and importance of reasonably allocating staff work hours. The weight analysis of the impact indicators showed that the employment rate and other labor market indicators were the main factors that encourage employees to work overtime, while social insurance labor protection factors had the smallest impact on an individual's work hours. Based on the conclusions of this study and the developmental experience of some developed countries, this study proposes the following suggestions in respect to the rational allocation of individual work hours.

### Accelerating the Reform of the Household Registration System and Promoting Integration of the Labor Market

Overtime work among the migrant workers group reflects the current segmentation of the Chinese labor market. Studies have shown that employment discrimination based on “hukou” is more serious, and it is also the main reason for the division of the urban labor market (118). Therefore, reform of the household registration system should be the main focus of attention in order to eliminate labor market segmentation and promote integration of the labor market, which would lead to a break away from the institutional separation of the market and a shift toward the establishment of a unified labor market in which labor can flow freely.

### Developing a Reasonable System Design and Promoting Work Sharing

The unemployment rate, wages and other factors have the highest impact on employee work hours. Moreover, some studies show that avoiding unemployment and increasing income level are the main reasons for employees' overtime work. While against the current backdrop of oversupply in the labor market, it is critical to urgently resolve how the income level of individuals can be ensured, particularly within the context of alleviating individual employment pressures. The implementation of work sharing is an important means of relieving the pressure on individuals in respect to their work hours, which can effectively maintain the national employment rate and reduce unemployment losses (119). Therefore, this study believes that seeking a reasonable system design and promoting the work sharing mechanism can not only liberate some employees who have been constrained by jobs for a long time, but also provide employment opportunities for more unemployed groups.

### Establishing and Improving the Information Disclosure System of Work Hours and Strengthening External Supervision

The research results showed that the social insurance coverage rate, medical insurance, and other factors that protect individuals' quality of health, as well as the employment rights of employees, had a low impact coefficient on individual work hours. On the one hand, the current government's protective measures for employees still need to be perfected, and regulations on overtime work and informal employment need to be established (120, 121). On the other hand, at present, China's domestic labor regulatory bodies are inadequate, and there is a lack in terms of the social supervision of industrial organizations, trade unions, Chambers of Commerce, and other associations (122). In response to the above situation, studies have shown that a timely and effective information disclosure system would not only provide a foundation and an important supplementary means of ensuring effective supervision (123), but it is also an important driving force that can improve the performance of organizations (124). Therefore, this study proposes that establishing and improving an information disclosure system of organizational work hours is an important means by which to effectively urge organizations to arrange work hours rationally, as well as strengthen the external supervision of organizational work hours. This system can, to a certain extent, regulate the work hours of employees, thus improving the quality of individuals' lives.

## Data Availability Statement

The raw data supporting the conclusions of this manuscript will be made available by the authors, without undue reservation, to any qualified researcher.

## Ethics Statement

Institutional review board approval was not needed because this was a database study and no participants were involved.

## Author Contributions

BL conceptualized this study, calculated the data, and drafted the article. HC designed the study and revised the article. XY polished this article. CH did valuable work on updating new literature and specifying research method in the process of modification.

### Conflict of Interest

The authors declare that the research was conducted in the absence of any commercial or financial relationships that could be construed as a potential conflict of interest.

## Supplementary Material

Supplementary Figure 1 The box plot of work hours.
