# Supplementary figures and images for: Why Work Overtime? A Systematic Review on the Evolutionary Trend and Influencing Factors of Work Hours in China
Source: Front Public Health. 2019 Nov 15;7:343. doi: 10.3389/fpubh.2019.00343 (PMC6872522; doi:10.3389/fpubh.2019.00343)

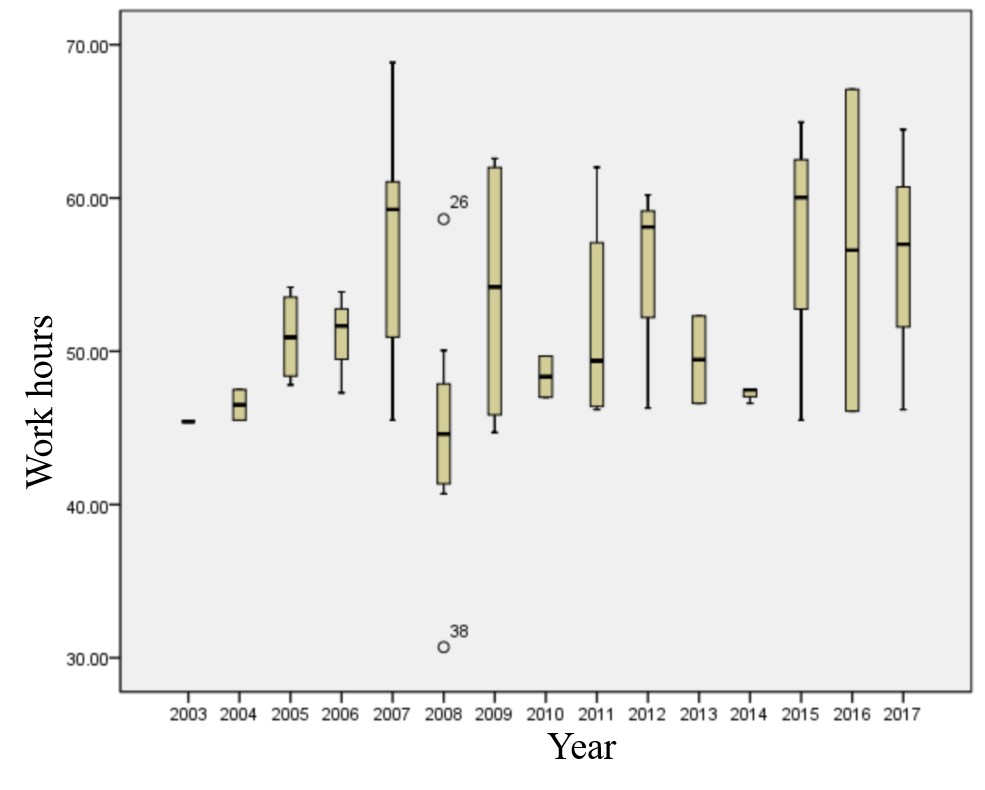

Supplement: Supplementary Figure 1 — The box plot of work hours. [file Image_1.JPEG]
